# Medical and Physical Intelligence

**Published:** 1805-02-01

**Authors:** 


### Medical and Physical Intelligence.

Dr. WALKER has enclosed in a letter to the Editors the following important communication:

" Dear Sir, *Bath, Jan.* 6, 1805.

" I shall be obliged by your communicating to the Medical Council of the Royal Jennerian Society the enclosed Resolutions of a meeting held here on Jan. 1, for the purpose of forming a Royal Somerset Jennerian Society. Our plan will include provision for corresponding with your Society, and making our returns to you.

Your very obedient servant,

THOMAS CREASER.

*To Dr. Walker, Salisbury Square, London.*

### Royal Somerset Jennerian Society.

Bath, Jan. 4, 1805.

At a numerous and highly respectable meeting, held on the 1st of January inst. at the Bath Agricultural Society's Room, to consider of the formation of A Somerset Jennerian Society for the extermination of the Small-pox, in pursuance of a requisition previously issued — Present, The Earl of Selkirk, Sir John Cox Hippisley, Bart. M. P. William Gore Langton, Esq. M. P. John Palmer, Esq. M. P. B. Hobhouse, Esq. M. P. Sir G. Colebrooke, Bart. Sir John Keane, Bart. Sir W. Watson, D. O. Parry Okeden, Esq. J. Ensor, Esq. John Wiltshire, Esq. Rev. Dr. Randolph, Dr. Haygarth, Dr. Crauford, Dr. Archer, Dr. Murray, Dr. Boisragon, Dr. Broderip, Dr. Moodie, J. Billingsley, Esq. Rev. —— Richards, Rev. R. Warner, Mr. Creaser, Mr. Tudor, &c. &c. Benjamin Hobhouse, Esq. M. P. in the Chair.

On the motion of John Wiltshire, Esq. it was resolved unanimously,

That the thanks of this meeting be transmitted through the Chairman to Dr. Jenner, expressive of the high sense which they entertain of his merit, and of the importance of his discovery.

That by the great diminution of deaths from Small-pox within the Bills of Mortality in London, (viz. from an average annually exceeding 2000 for the last fifty years of the past century, to the number of 536 within the year ending Dec. 1804, being the period of the greater part of the existence of the Royal Jennerian Society) it appears to this Meeting, that by the universal adoption of similar measures, there is a certain prospect of the speedy extermination of Small-pox.

That this Meeting do form itself into a Society, to be called *The Royal Somerset Jennerian Society*, for the extermination of the Small-pox.

That a subscription be opened to prosecute with vigour these salutary intentions; and that five guineas at one payment, or one guinea annually, shall constitute a governor.

That this Meeting do, through the Chairman, request His Royal Highness the Duke of York to honour the Society with his name and support as Patron.

That this Meeting do nominate and appoint the Right Hon. Earl Poulett to be President, and his Grace the Duke of Bedford, the Right Hon. Lord Somerville, the Members for the County of Somerset, the Members for the City of Bath, Sir John Cox Hippisley, Bart. Sir G. Colebrooke, Bart. Sir W. Watson, B. Hobhouse, Esq. M. P. John Wiltshire, Esq. Col. Strode, Col. Leigh, the Mayor of Bath, and the Rev. Dr. Randolph, to be Vice-Presidents of this Society, and that the number of Vice-Presidents shall not be less than twenty-four.

That this Society do appoint C. Phillot, Esq. Treasurer.

That a Committee be now appointed, and that H. White, Esq. J. Horton, Esq. C. Crook, Esq. D. O. Parry Okeden, Esq. John Ensor,

Ensor, Esq. Dr. Parry, Dr. Haygarth, Dr. Archer, Dr. Gibbes, Dr. Crawford, Dr. Murray, Dr. Boisragon, Dr. Brodcrip, the Rev R. Warner, Mr. Creaser, Mr. Tudor, be Members of the said Committee, which shall not be less than twenty-four in number; that all Vice-Presidents shall be Members of the said Committee, and the said Committee be appointed for the purpose of considering and carrying into effect the important objects of this Meeting; that three of the said Committee shall form a Quorum, and shall be enabled to draw on the Treasurer for such sums as may be necessary, and shall report all its proceedings to another General Meeting.

That subscriptions be received at Messrs. Hobhouse and Co's Bank.

That the Physicians and Surgeons of the Casualty Hospital and Bath City Dispensary be invited to co-operate with the views of this Society.

On the motion of Sir John Cox Hippisley, it was resolved unanimously,

That a Copy of these Resolutions be sent to the Chairman of the next General Quarterly Sessions, and also to the Grand Jury, at the ensuing Lent Assizes for this County, for their approbation and support.

On the motion of the Rev. Dr. Randolph, it was resolved unanimously,

That the Bishop of this Diocese be respectfully informed of the Resolutions of this Meeting, and requested to recommend the objects of it to the different parishes in his Diocese.

On the motion of John Wiltshire, Esq. seconded by J. Palmer, Esq. it was resolved unanimously,

That the thanks of this Meeting be given to B. Hobhouse, Esq. for his able, eloquent and impressive Address from the Chair; and to Mr. Creaser, for his unremitted and important services towards the formation of this Society.

Signed B. Hobhouse, Chairman.

---

### Vaccine Inoculation.

*Statement of the Number of Persons inoculated at the Stations of the Royal Jennerian Society, in eighteen Months, from the Quarterly Reports.*

| | | | |
|---|---|---|---|
| Central House - - - | 2911 | Bishopsgate - - - | 1070 |
| Surry Chapel - - - | 2110 | Hoxton - - - - | 816 |
| Maze Pond, Southwark | 387 | Golden Lane - - - | 579 |
| Rotherhithe - - - | 510 | Clerkenwell - - - | 245 |
| Shadwell - - - - | 512 | Gate Street, Holborn | 216 |
| Mile End - - - - | 516 | Mary-le-Bone - - - | 1523 |
| John Street, Minories | 400 | Westminster - - - | 218 |
| Inoculated before the Central House was opened - | | | 275 |
| | | Total - | 12288 |

N.B. In the same period, 19,352 charges of vaccine virus have been supplied from the Central House, in Salisbury Square, free of expence, to applications from most parts of the British empire, and foreign countries.

---

It will doubtless be highly gratifying to the public, to observe the remarkable decrease of deaths by the small-pox, as appears by the following comparative view, extracted from the Bills of Mortality:

| | 1803 | 1804 |
|---|---|---|
| January | 181 | 120 |
| February | 121 | 77 |
| March | 95 | 44 |
| April | 61 | 38 |
| May | 69 | 88 |
| June | 48 | 29 |
| July | 50 | 35 |
| August | 67 | 27 |
| September | 85 | 33 |
| October | 64 | 50 |
| November | 152 | 45 |
| December | 180 | 50 |
| Total | 1173 | 586 |

This decrease will appear still more important when compared with the annexed statement of deaths by small-pox, for fifty years, within the Bills of Mortality, averaged by ten years.

| From | to | |
|---|---|---|
| 1750 | 1759 | 19,642 |
| 1760 | 1769 | 24,435 |
| 1770 | 1779 | 22,039 |
| 1780 | 1789 | 17,121 |
| 1790 | 1799 | 17,685 |
| | Total | 100,922 |

Making an annual average of 2018 deaths per small-pox in fifty years.

The following is an annual statement of deaths in the present century:

| | |
|---|---|
| 1800 | 2409 |
| 1801 | 1461 |
| 1802 | 1579 |
| 1803 | 1173 |
| 1804 | 586 |

It is hoped the knowledge of these facts will be strongly promotive of the beneficial practice of vaccine inoculation, it appearing that the fatal disease of small-pox has progressively declined as the inestimable discovery of Dr. Jenner has been introduced.

---

A Cor-

A Correspondent, signed W. S. Hanslope, December 4, 1804, in a letter to the Editors, writes thus:

"As you occasionally admit into your Journal criticisms on medical words, no apology seems necessary for addressing to you the following communication.

"In the language of medical men themselves, MANMIDWIFE is the term applied to those who practise the obstetric art; but which is so indelicate, uncouth, and barbarous, as to be highly exceptionable. In the present advanced state of science and refinement of language, surely some other term, more appropriate and decorous, might be contrived to answer the same purpose. I will venture to propose one purely English. The OPUS OBSTETRICIS is termed a delivery; to substitute DELIVERER for MANMIDWIFE might not therefore be improper. This may not indeed be considered by the faculty as sufficiently technical; I have no doubt, however, but some of your intelligent Correspondents will be able to supply the *desideratum;* and then we may hope to see the present hermaphrodite expression consigned to merited oblivion.

---

The following extract from the circular letter addressed to the physicians of Public Institutions in London, will shew the zeal and activity of the Society for bettering the condition of the poor.

"Another desirable object of the Committee will be to form, in conjunction with the parishes in the Bills of Mortality, such a parochial plan for preventing the introduction of infectious fever within their respective limits, as may hereafter be adequate to the object of preventing the renewal and prevalence of febrile infection in the Metropolis, without any further call for Parliamentary aid.

"With these views, the Committee solicits assistance and information, for the attainment of an object, which is important, not only to all classes of persons in the Metropolis, but to the country at large.—The enclosed queries will shew the nature of the information to which we request your early attention; hoping that, by a speedy answer, you will favour the Committee with assistance, in forming its plan of operation.

We have the honour to be,
Your faithful and obedient Servants,

S. DUNELM. W. WILBERFORCE.

SOMERSET. N. VANSITTART.

GEORGE ROSE. T. BERNARD.

R. PEELE.

"QUERIES from the Select Committee for preventing the spreading of contagious malignant Fevers in the Metropolis.

"1. What are the streets, courts, alleys, and other places in your neighbourhood where infectious fever prevails?

"2. How

"2. How long has the infection continued in any such place respectively?

"3. What means have been adopted by the parish, or neighbourhood, for curing such fever, or for preventing the spreading of the infection?

"4. How far can the Select Committee be useful in assistance towards such an object?

"5. How far do you think your parish, or any hospital in your neighbourhood, will be disposed to further the objects of the Select Committee?

"6. Have you any other observations or suggestions to offer to the Select Committee?"

"*Please to address your answers to the* SELECT COMMITTEE *for* PREVENTING CONTAGIOUS FEVERS, *No.* 190, *Piccadilly.*

---

A Pamphlet has been sent to us, attempting to demonstrate that the grease in horses is the same as scrophula in the human subject. It has been already suggested, that it would be *infra dignitatem* to pay any attention to such a production, and therefore we decline troubling our readers with any further notice of it.

---

Mr. CHARLES BELL has submitted a proposal to the Managers of the Royal Infirmary of Edinburgh, for the establishment of a Museum under the patronage of the Hospital; with considerations on the means of improving the knowledge of morbid Anatomy. In this address Mr. B. endeavours to impress the Managers with the utility of such an establishment, and the ease with which it may be formed. On both these points, we suppose, there will be little difference of opinion; but we doubt much whether this pamphlet will in any degree contribute towards the end proposed. The "war of passion and prejudice," which delayed this proposal, will doubtless be rekindled by the terms which it implies.

---

*Lecture Room, No. 18, Wilmott Street, Russell Square.*

Mr. MILBURNE's Lectures on Physiology, or Doctrine of the Animal Economy, will commence on Wednesday evening, the 20th inst. and be continued every succeeding Wednesday and Saturday evening, at eight o'clock precisely; the course will be illustrated by anatomical preparations, drawings, prints, &c. &c.

Further particulars may be known by applying to Mr. M. at the Lecture Room.

---

Mr. MOOR, Surgeon Dentist to Her Royal Highness the Duchess of York, will commence his Spring Lectures on the Structure and Diseases of the Teeth, on February the 14th, at his house, No. 6, Palsgrave Place, Temple.

SIR

Sir John Sinclair, whose unwearied industry on every subject to which he turns his attention must command the gratitude and admiration of the public, is at present engaged in the composition of a work, founded on experience and a system of extensive enquiry, to be entitled, "The Code of Health and Longevity." The patriotic author proposes to divide his work into the three following parts: 1. The circumstances which necessarily tend to promote health and longevity, independent of individual attention. 2. The rules which, if observed by an individual, have a tendency to preserve health and existence, even where these independent circumstances are wanting. And, 3. The regulations by which the general health and safety of a great community are protected from the various injuries to which they are likely to be exposed.

Messrs. A and C. R. Aikin have, for a considerable time, been preparing for publication a Dictionary of Chemistry and Mineralogy, with their application to Arts and Manufactures. This work has been some time in the press; it will be be comprised in two quarto volumes, and be published with expedition.

Dr. Clutterbuck is about to publish an Inquiry into the Seat and Nature of Fever, as deducible from the Phenomena of the Disease, and the Principles of the animal Economy; in which he hopes to determine more satisfactorily than has been hitherto done, some disputed points of this long contested subject.

Dr. Stock, of Bristol, has in the press a work, entitled, Medical Collections on the Effects of Cold as a Remedy in certain Diseases.

Dr. Thornton will publish, in a short time, an Answer to the various Objections raised against Vaccination, with Proofs of the Efficacy of the Cow-pock, intended principally for the use of Families.

---

The Editors think it a duty which they owe themselves and the Medical World to state, that a number of Actions have been commenced against their Printer and different Booksellers, by an Attorney of the name of Smith, of Robert Street, Adelphi, at the Suit of "William Brodum, Esquire," for the publication of the paper which appeared in the last Number of this Work, on the subject of Quacks and Empiricism. The Trials of these Actions are expected to come on, in the Court of Common Pleas, about the middle of February.

---

